# Heterogeneous Antibiotic Resistance Gene Removal Impedes Evaluation of Constructed Wetlands for Effective Greywater Treatment

**DOI:** 10.3390/antibiotics13040315

**Published:** 2024-03-29

**Authors:** Daniella Itzhari, Weitao Shuai, Erica M. Hartmann, Zeev Ronen

**Affiliations:** 1 Zuckerberg Institute for Water Research, The Jacob Blaustein Institutes for Desert Research, Ben Gurion University of the Negev, Beersheba 8499000, Israel; vanderro@post.bgu.ac.il; 2 Department of Civil and Environmental Engineering, Northwestern University, Evanston, IL 60208, USA; weitao.shuai@northwestern.edu (W.S.); erica.hartmann@northwestern.edu (E.M.H.); 3 Center for Synthetic Biology, Northwestern University, Evanston, IL 60208, USA; 4 Division of Pulmonary Medicine, Department of Medicine, Feinberg School of Medicine, Northwestern University, Chicago, IL 60611, USA

**Keywords:** greywater reuse, antimicrobial resistance genes, qPCR, metagenomic sequencing

## Abstract

Microorganisms carrying antimicrobial resistance genes are often found in greywater. As the reuse of greywater becomes increasingly needed, it is imperative to determine how greywater treatment impacts antimicrobial resistance genes (ARGs). Using qPCR and SmartChip™ qPCR, we characterized ARG patterns in greywater microbial communities before, during, and after treatment by a recirculating vertical flow constructed wetland. In parallel, we examined the impact of greywater-treated irrigation on soil, including the occurrence of emerging micropollutants and the taxonomic and ARG compositions of microbial communities. Most ARGs in raw greywater are removed efficiently during the winter season, while some ARGs in the effluents increase in summer. SmartChip™ qPCR revealed the presence of ARGs, such as tetracycline and beta-lactam resistance genes, in both raw and treated greywater, but most abundantly in the filter bed. It also showed that aminoglycoside and vancomycin gene abundances significantly increased after treatment. In the irrigated soil, the type of water (potable or treated greywater) had no specific impact on the total bacterial abundance (16S rRNA gene). No overlapping ARGs were found between treated greywater and greywater-irrigated soil. This study indicates ARG abundance and richness increased after treatment, possibly due to the concentration effects of the filter beds.

## 1. Introduction

Greywater reuse is increasing globally; however, treatment systems are ineffective in removing all contaminants. Treatment systems are usually designed to remove nutrients and organic matter rather than emerging micropollutants, antimicrobial-resistant bacteria, and antimicrobial-resistance genes (ARGs). This issue is all the more pressing as emerging micropollutants and ARGs in greywater treatment systems are interrelated [1]. Treatment systems provide an optimal environment for developing resistance, as exposure to emerging micropollutants may enrich ARGs [2,3]. Greywater treatment systems have a high potential to remove ARGs (up to 97%) [4], but performance varies wildly with the conditions and technology [5].

The term “antibiotic” has been used to characterize molecules that inhibit or kill microorganisms, classified based on their chemical structure or mechanisms of action [6]. Residues of antibiotic compounds can impose pressure on the wastewater microbiome, even in concentrations below minimal inhibitory concentrations (MIC) [7]. In this article, emerging micropollutants are defined as a group of compounds, including toxic nanoparticles, personal care products, heavy metals, and microplastics. Recent research identified the occurrence of bacteria and emerging micropollutants in household greywater as a potential driver of the accumulation of antimicrobial-resistant bacteria [8,9,10]. Emerging micropollutants can act in an unspecific manner, targeting different sites or processes and causing harm to bacterial cells [11,12]. Although this mode of action differs from antibiotics that attack specific targets, the same protective mechanisms against antibiotics may also confer tolerance to emerging micropollutants. Constructed wetlands provide a low-cost solution for greywater treatment regarding removing nutrients, bacteria, pathogens, and ARGs. Constructed wetlands have been employed to remove antibiotic residues and emerging micropollutants [13]. Given the benefits of ARG removal, low construction costs, maintenance, energy requirement, and performance efficiencies, constructed wetlands offer a promising solution for developing/low-income countries.

The occurrence and fate of emerging micropollutants in constructed wetland systems have only recently garnered attention, and more studies are needed to understand their fate in these systems comprehensively [14,15]. The most detected emerging micropollutants in greywater are triclosan (biocide), methyl- and propylparaben (preservatives), galaxolide and tonalide (fragrances), oxybenzone and octocrylene (U.V. filters) [16], and benzalkonium chloride (biocide) [17]. The removal mechanisms for emerging micropollutants in constructed wetlands are complicated and typically ascribed to plant uptake, substrate sorption, and microbial biodegradation [18,19]. Although emerging micropollutants may be toxic to microbes, biodegradation is still the dominant process for their removal [20,21]. The removal efficiencies of emerging micropollutants in constructed wetlands varied between 7 and 87% for triclosan [22,23], while for galaxolide, it was reported to fluctuate between 42 and 95% [22,24]. Due to its chemical structure, galaxolide is barely biodegradable and is removed mainly by sorption, which can occur in municipal wastewater treatment plants or constructed wetlands [25,26]. High temperature and intense sunlight irradiation enhanced the activities of plants and microorganisms in constructed wetlands, resulting in increased elimination of emerging micropollutants [23].

An example of an efficient wetland system is a Recirculating Vertical-Flow Constructed Wetland (RVFCW) (Figure S1, Supplementary Materials). RVFCW systems efficiently remove 90–100% of the suspended solids, reducing biological oxygen demand (BOD) by 95–100% and about 70% of total nitrogen [27,28]. However, although fecal coliforms decreased by 99%, both *Staphylococcus aureus* and *Pseudomonas aeruginosa* could be found in most treated greywater samples in which *E. coli* was reduced to non-detectable levels [29]. In addition, *Pseudomonas putida* displaying clinical resistance to cephems and carbapenems was identified in treated greywater [30]. Thus, while otherwise efficient, constructed wetland treatment systems are potential hotspots for the spread of ARGs [31].

ARGs can be removed via biodegradation of the genetic material, antibiosis, adsorption, and filtration [32], of which biodegradation is the primary pathway [33]. Antibiosis is the production of specific antibiotics by fungi or bacteria to inhibit the growth of competing bacteria and thereby decrease ARG abundance [34]. Similarly, plant roots can release antibiotic compounds. For example, the plant *Melaleuca ericifolia* inhibits the growth of *E. coli* [35,36]. However, plant-mediated antibiosis is unlikely to be the major driver of ARG removal in RVFCWs, as it has been reported that effluents of the RVFCWs were of high quality even when operated without plants in the filter bed [29]. Alternatively, adsorption and filtration are physical processes to remove bacteria and ARGs in wastewater [37]. In both mechanisms, the type of filter medium plays a fundamental role [38,39]. For example, zeolite and gravel filtration resulted in up to 50% removal of *tet* genes [40] and up to 78% of antimicrobial-resistant bacteria [41].

Inefficient removal of emerging micropollutants and ARGs in constructed wetland systems is alarming, especially since irrigation with treated greywater is an increasing practice. Their release into the environment leads to unpredictable long-term consequences, and their presence in the soil, even at very low levels, may pose a risk to human health [42,43,44]. Once in the soil, the fate and behavior depend on various chemical, physical, and biological processes [45], which may affect the soil microbial community [46]. Nevertheless, recent studies demonstrated that irrigation with treated greywater does not seem to impact ARG levels in the soil microbiome [7,47].

The importance of this study arises because of the increasing discharge of treated greywater into the environment. Most studies have focused on detecting and removing ARGs in treated greywater. However, a thorough analysis of the fate of ARGs and emerging micropollutants from treated greywater in the soil has yet to be determined. In this context, this research aims to characterize the prevalence of ARGs inside constructed wetland systems and soil. Based on qPCR and SmartChip™ high-throughput qPCR, our specific objective was determining the resistome in different parts of the RVFCW system and irrigated soil. We present results of the identification and characterization of ARGs from the filter bed, raw and treated greywater, together with samples of soil irrigated with treated greywater or potable water. Although residual levels of emerging micropollutants and ARGs have been detected in treated greywater-irrigated soils in previous research, the presence of these elements has not been linked to microbial community composition. Therefore, we present the metagenomics results of these soil samples, providing a comprehensive observation of the irrigated soil microbial community.

## 2. Results and Discussion

### 2.1. Physicochemical Analysis of Influent and Effluent

Raw greywater was sampled twice in successive months during the year 2021, and treated greywater was sampled during 2015–2021. All samples were analyzed for physicochemical parameters that might impact the microbial community (Table 1). The most critical factors for removing bacteria are temperature, biological oxygen demand (BOD), and pH [48]. Mean pH values of raw greywater ranged from 6.95–9.63 and 6.1–10.16 for treated greywater. Microbial growth and survival are optimal at a pH of 5.5–7.5 [49] and decrease at higher pH. The BOD level can be used as an indicator for the decomposition of organic compounds by aerobic microorganisms, i.e., a decline in BOD levels reflects a decay in organic compounds [50]. Our results showed that the BOD levels ranged from 10.9–39.9 in raw greywater and 0.45–21.9 in treated greywater. Hence, organic matter was degraded during treatment.

Overall, the values of the treated greywater did not vary significantly among the different households and during the years, meaning that the systems were stable and well-established (See Table S2, Supplementary Materials). Based on Table 2, it can be concluded that the RVFCW system effectively removes chemical and biological contaminants. Overall, it reduces BOD and TSS by 80%, TOC by 60%, TN by 30%, and turbidity by 90%. These findings corroborate previous results [29], reconfirming that the RVFCW is a promising treatment system for greywater use.

#### 2.1.1. Performance of RVFCW for ARG Removal from Greywater

**Figure 1:**
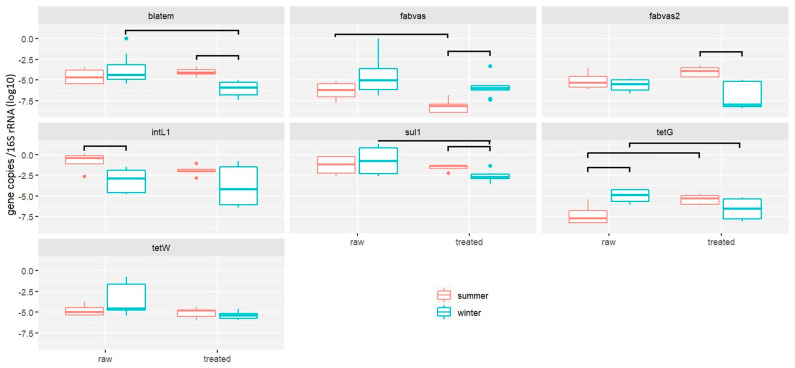
Relative abundance of antimicrobial resistance genes in greywater samples collected from different families. Data are shown on a log scale as relative gene copies normalized to the copies of 16S rRNA genes from each sample (*n* = 16 for winter, *n* = 13 for summer). Box plot explained: The lower and upper sides of the box correspond to the first and third quartiles. The vertical line that split the box in two is the median. The whiskers extend from the box to either the smallest or largest value. Outlying points are plotted individually (dots).

A total of 29 greywater (raw (*n* = 14) and treated (*n* = 15)) samples were collected during the years 2021 and 2022. The relative abundance of ARGs in these samples was analyzed via quantitative PCR (Figure 1). Relative gene abundance was calculated by normalizing the absolute number of ARG copies to 16S rRNA gene copies [51]. The absolute 16S rRNA gene copy number did not change significantly after greywater treatment (*t*-test, *p* = 0.12), in accordance with a previous study [52]. During summer, a significant decrease was detected in the gene *fabVAS2* (*p* = 0.033), while gene *tetG* increased after treatment (*p* = 0.02). In the winter season, most of the genes of interest significantly decreased after treatment, specifically *tetG* (*p* = 0.027), *sul1* (*p* = 0.005), and *blaTEM* (*p* = 0.002). Overall, the RFVCW system removes most ARGs efficiently during the winter season, which may be attributed to multiple factors [47], such as the anaerobic and aerobic processes within the treatment system [53] or the removal of biomass or solids [54]. In treated samples, a significantly higher abundance after treatment was detected for genes *sul1*, *blaTEM*, and *fabVAS2* during summer. Only *fabVAS* was significantly lower during summer compared to treated samples. In raw samples, *tetG* was significantly higher in winter, while *intI1* was higher in summer. When comparing the relative gene copy abundance without taking into consideration the season, we found no significant difference between raw and treated samples of greywater (see Figure S2, Supplementary Materials).

Typically, the highest relative abundance was detected for *intI1*, which agreed with a previous study [55]. The abundance of *intI1* is known to alter rapidly in natural reservoirs owing to the short generation time of host cells and gene transfer mechanisms, and thus, is a suitable marker of the pollution level in the environment [56].

#### 2.1.2. Quantification of Antimicrobial Resistance Genes by Resistomap

**Figure 2:**
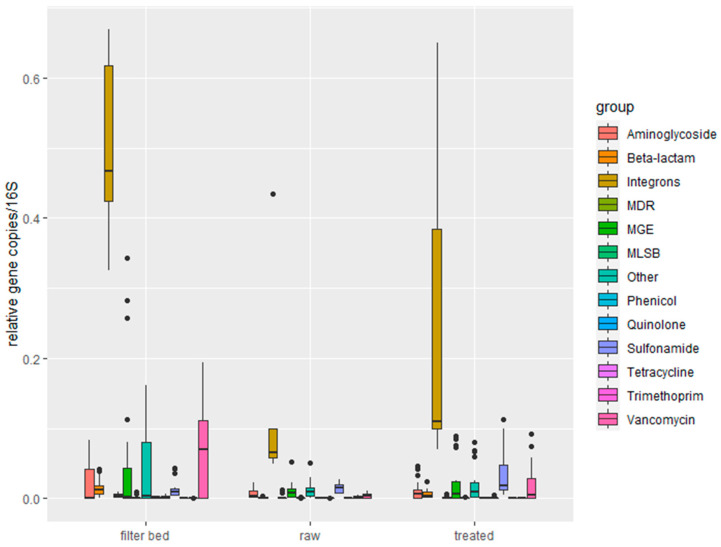
ARG relative abundance normalized to 16S rRNA genes, MDR = Multi Drug Resistance, MGE = Mobile Gene Element, MLSB = macrolide, lincosamide and streptogramin B. See Figure 1 for box plot statistics.

We detected 34 genes in the raw greywater, treated greywater, and the filter bed of the RVFCW system (Figure 2). The vancomycin resistance genes *vanXB* and *cmr* gene were not detected in any sample. The latter is associated with resistance efflux pumps and has been reported to be linked to antibiotic resistance induced by antimicrobial chemicals [10]. The multidrug–efflux resistance gene *ermD* and tetracycline resistance gene *tetC* were detected only in filter bed samples. The relative abundance of ARGs for each sample, clustered according to each household, is shown in Figure S3 Supplementary Materials. The average absolute abundance of ARGs was 2.18 × 10^5^/mL raw greywater and 1.56 × 10^5^/mL treated greywater, respectively, and 2.98 × 10^5^/g of the filter bed. Average 16S concentrations were 2.49 × 10^7^, 1.10 × 10^7^, and 1.56 × 10^7^ of raw, treated greywater, and filter bed samples, respectively. Therefore, the relative average abundances of ARG to 16S in the raw greywater, treated greywater, and filter bed samples are 8.74 × 10^−3^, 1.42 × 10^−2^, 1.91 × 10^−2^, respectively. Thus, filter bed samples had the highest concentrations of ARGs per 16S rRNA gene copy. Comparing the average relative ARG abundance after the treatment, we see an increase in 10 genes: *vanA; mcr1_1*; *fabK*, *sul2_1*; *aph3-ib*; *blaCTX-M*; *blaOXA51*; *qnrB*; *ermF*; and *tetW*. Those genes confer resistance to vancomycin, aminoglycosides, beta-lactams, and quinolones.

It is notable to mention that distinct ARGs appear in different households, and even if samples originate from the same household, they are distinct at other locations of the RVFCW. Regarding the relative abundance, 64% percent comes from the filter bed. Treatment systems of greywater may increase the abundance of ARGs rather than remove them since they exist in sludge [57]. Table 2 indicates the highest detected gene abundance and its origin.

**Figure 3:**
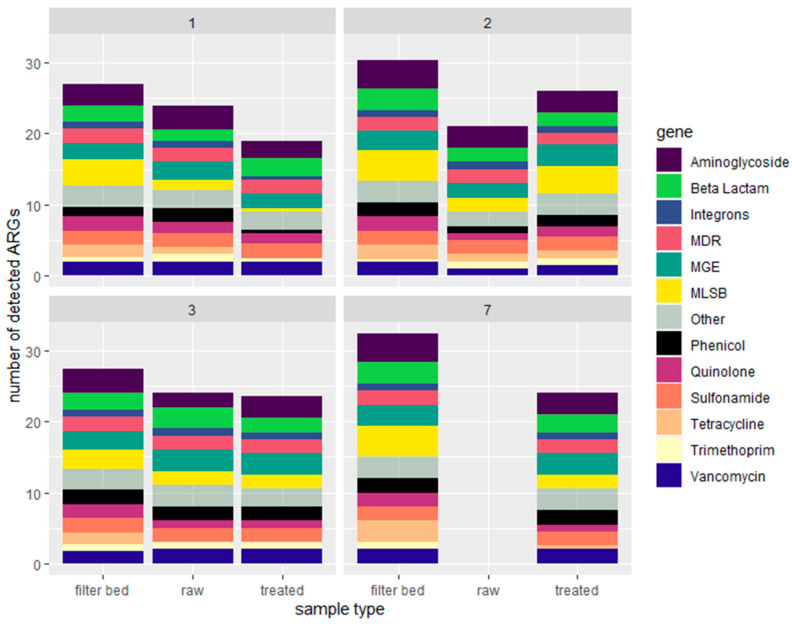
The average number of ARGs (richness) divided per household (the household number written in the heading), as detected by resistomap in raw greywater, treated greywater, and filter bed. MDR = Multi Drug Resistance, MGE = Mobile Gene Element, MLSB = macrolide, lincosamide and streptogramin B. See Figure 1 for box plot statistics.

When dividing the ARGs into the different resistance classes, the most occurring resistance mechanism is the efflux pump, as we also found previously [52]. Resistance genes involved in efflux comprised 12–96% of the ARG abundance for individual greywater samples, which summed up to 48% of the total ARG abundance for all (raw and treated) samples. The observed increase in ARGs after treatment differs from the values reported for decentralized greywater treatment systems. The average number of ARG detected in raw and treated greywater is 23, and the filter bed is 29 (Figure 3).

Among the different genes monitored in this study, the number of integrons (*intI1*) was the highest in all the samples. As mentioned before, the *intI1* gene indicates anthropogenic pollution [58] and is also associated with environments polluted with disinfectants/biocides and quaternary ammonium compounds [59]. The genes *tetW*, *tetA_1*, and *tetC_1*, coding for tetracycline resistance, were only detected in the filter bed samples. Interestingly, new ARGs may occur in the bacterial population even without antibiotic exposure, as is the case for tetracycline-resistant genes [60]. The resistance of the tetracycline family is a natural phenomenon that gives bacteria adaptive advantages for obtaining resources in the environment compared to other competing species [32]. In addition, tetracycline-resistant genes are highly likely to be transferred to indigenous environmental bacteria and can migrate quickly to the surface of biofilms, especially gene *tetW* [53].

**Figure 4:**
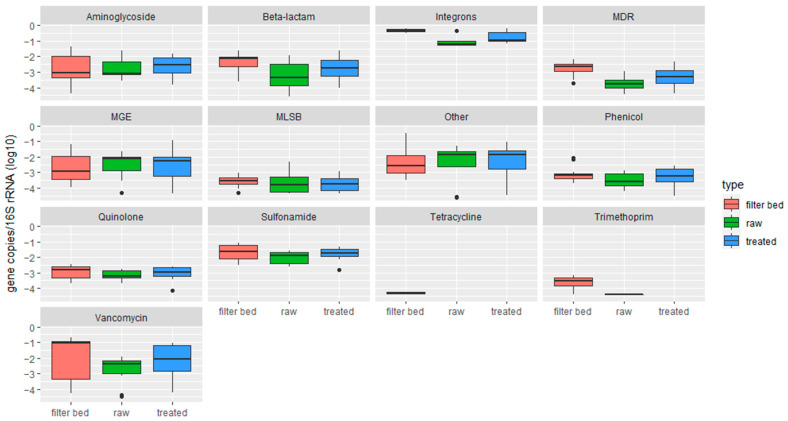
Relative abundance of ARGs normalized to 16S rRNA gene copies of untreated (raw) and treated greywater and filter bed. The abundance is on a log10 scale and relative to the 16S rRNA gene copy number. See Figure 1 for box plot statistics.

ARG abundance increased after treatment (Figure 4), specifically in the ARG classes vancomycin, sulfonamide, aminoglycoside, beta-lactam, MDR, quinolone, and phenicol. However, only aminoglycoside and vancomycin gene abundances significantly differ between raw and treated greywater (*p* = 0.037 and *p* = 0.0373, respectively).

In all households, we observed a significant (*p* < 0.05) increase in the treated greywater samples in ARG relative abundances normalized to the 16S rRNA gene. Bacteria retained inside the filter beds could be the explanation for this observation. RVFCW can be considered a biofilm-based wastewater treatment system; environmental biofilms are reservoirs of ARGs. Thus, a concentration effect within the system is a possible explanation for tetracycline-resistant bacteria in the treated water [61]. In contrast, however, comparing the abundances of ARGs in the filter bed and a trickling filter suggested that there was no difference in the prevalence of ARG mobilization in the treated effluents [62]. The presence of ARGs, such as tetracycline and beta-lactam resistance genes, have been detected in both raw and treated greywater, but most abundantly in the filter bed (Figure S4, Supplementary Materials). Like other biological wastewater treatment systems, the RVFCW system does not remove all the ARGs from greywater. Moreover, it sometimes increases the abundance of ARG, as ARGs exist in sludge [63].

### 2.2. Characterization of Soil Samples and Quantification of Detected ARGs

**Figure 5:**
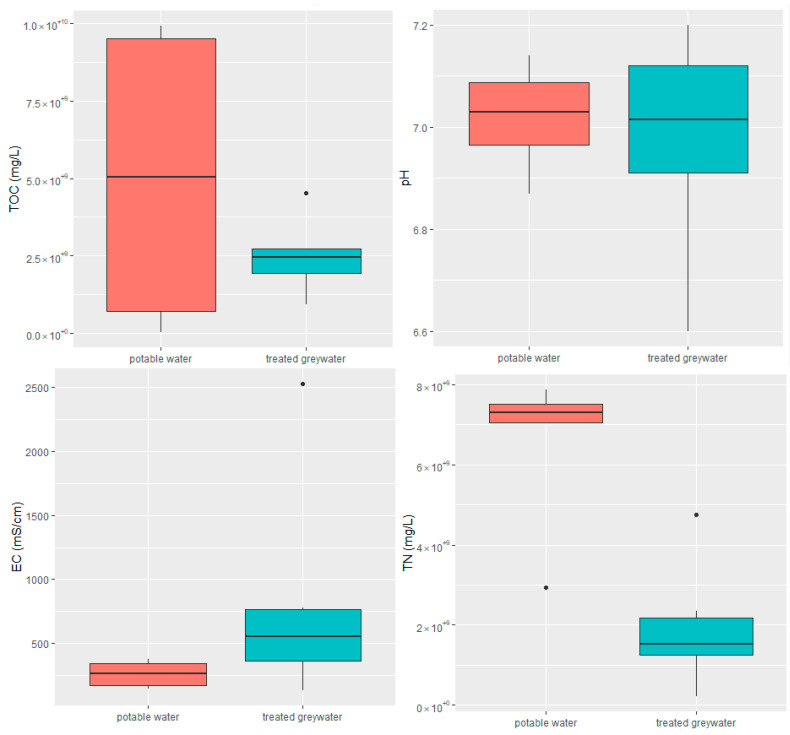
Physiochemical parameters of soil samples irrigated with treated greywater or potable water. EC—electrical conductivity; TOC–total organic carbon; TN—total nitrogen. See Figure 1 for box plot statistics.

Thirteen soil samples (irrigated with potable water (*n* = 7) or treated greywater (*n* = 6)) were collected during 2021 before the rainy season. The physicochemical parameters of the soil samples are shown in Figure 5. TOC and TN values of soil irrigated with potable water were significantly lower than those of soil irrigated with treated greywater (*p* = 0.0038 and *p* = 0.000781, respectively). Typically, treated greywater has a higher load of organics and chemical pollutants, contributing to higher values of EC than potable water. The mean pH of the soil did not differ significantly between potable water and treated greywater, which was important to notice since pH might affect the relative abundances of ARGs in the soil [64].

**Figure 6:**
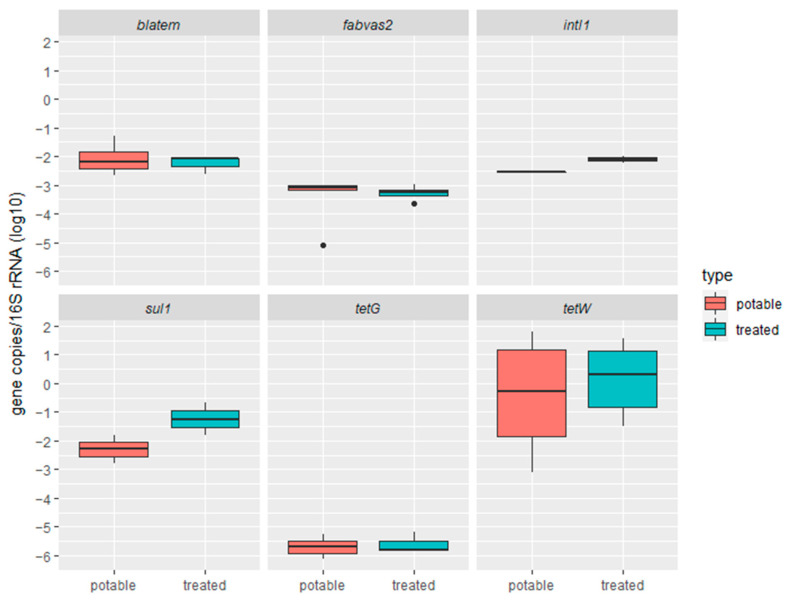
Relative abundance of antimicrobial resistance genes in soil samples collected in gardens irrigated with potable water or treated greywater. Data are shown on a log scale as relative gene copies normalized to the copies of 16S rRNA gene from each sample (*n* = 13). See Figure 1 for box plot statistics.

Only six of the seven genes targeted in the qPCR analysis were detected (Figure 6). The type of irrigation water did not influence the total bacterial abundance (16S rRNA gene) in the soil. The highest ARG relative abundance was detected for *tetW*, the most widely spread tetracycline resistance gene class [65]. Reports worldwide indicate that irrigation with treated greywater presents no greater risk than irrigation with potable water [66], assessing the survival of treated greywater-associated bacterial pathogens and ARGs in irrigated soils [67]. While many ARGs were present in treated greywater, no significant differences were observed between soils irrigated with potable water or treated greywater (see Table S3 Supplementary Materials). To give another example, irrigation with treated wastewater does not seem to impact the level of ARGs in the soil [68]. However, these approaches only target a few well-studied resistance genes [69]. A study focusing on the abundance of 147 genes detected an enrichment (up to 1000-fold) of ARGs compared to unamended soils [70]. The effect of irrigation with treated greywater on antibiotic resistance in the soil was variable among the studies [71], highlighting the need to understand better to what extent ARGs are disseminated.

#### 2.2.1. Analysis and Quantification of Emerging Micropollutants in the Soil

**Figure 7:**
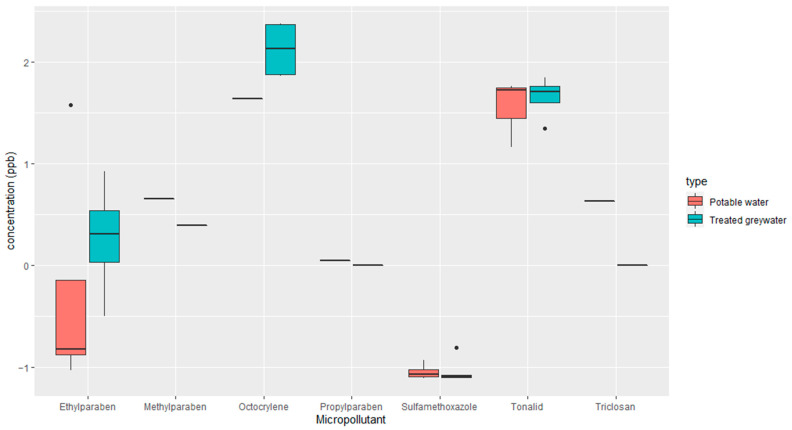
Concentrations of emerging micropollutants (ppb on the log scale) detected in potable water or treated greywater irrigated soil. See Figure 1 for box plot statistics.

The introduction of emerging micropollutants into the soil and their effect on ARGs in the soil microbiome have yet to be studied. Based on the results, micropollutants are present in the soil, but only octocrylene appears to be coming from the RVFCWs (Figure 7). Remarkably, tonalid was detected in the soil at a relatively high concentration, even when irrigated with potable water. This is interesting, especially since some emerging micropollutants, such as methylparaben, have been reported to be of natural origin and not only synthetic [72]. In contrast, tonalid in trace concentrations could indicate human activity [73]. The behavior of emerging micropollutants in agricultural soils is complex and depends on several factors, such as the type of compound and their physicochemical characteristics [74]. The accumulation of emerging micropollutants in soil can lead to an imbalance of microorganisms and a reduction in agricultural production efficiency [75,76]. Some studies have indicated that some emerging micropollutants in agricultural soil could reduce the diversity of the microbial community, ultimately change its structure [77,78], and increase the prevalence of antimicrobial-resistant bacteria in soil microcosms [79].

#### 2.2.2. ARG Identification through Short Read-Based Metagenomic Analyses

**Figure 8:**
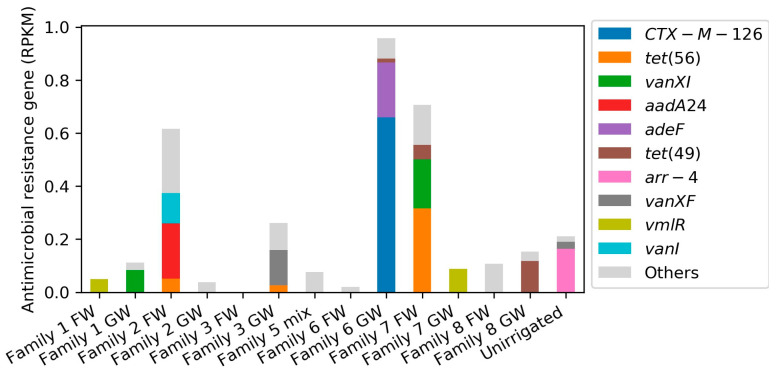
Top 10 antimicrobial resistance genes (ARGs) identified in soil samples from short read-based analyses, based on relative abundance in RPKM. FW-soil irrigated by potable water; GW-soil irrigated by treated greywater; unirrigated soil as the control sample.

Across all households, 26 different ARGs were identified in soils irrigated with potable or treated greywater, with 28% of the ARGs detected in one individual sample and *tet(56)* occurring the most frequently. Only two ARGs were identified in the unirrigated control sample. In addition, soil and greywater samples showed very different sample characteristics. The ARGs’ cumulative relative abundances (RPKM) from the short read-based RGI output showed no specific trend in soils irrigated by potable water or treated greywater in the same household (Figure 8), while both richness and cumulative relative abundances of ARGs showed descending trends after treatment in greywater samples [52]. ARG composition possibly highly depends on distinctive environmental factors in each household rather than the water supply [80]. For example, a study by Wang et al. revealed by metagenomic profiling that ARGs in emerging micropollutants contaminated soils were approximately 15 times more abundant than those in the less-contaminated ones [81].

**Figure 9:**
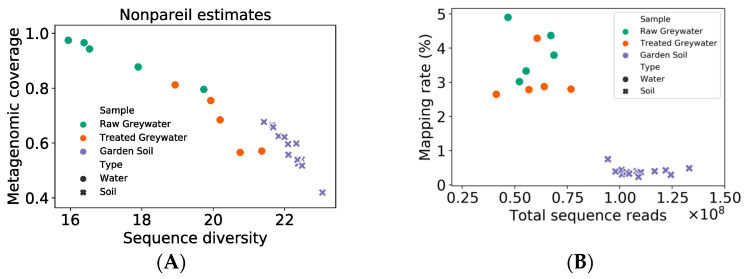
Nonpareil estimated metagenome coverage and diversity (**A**) and percentage of reads mapped to CARD ARGs (**B**). Nonpareil applies a redundancy-based approach to provide an abundance-weighted coverage of the metagenome, representing the fraction (range 0–1) of the microbial community sampled by DNA sequencing.

It is worth noting that the characteristics of metagenomes are very different for soil and greywater samples. Soil samples showed lower metagenomic coverage and higher sequence diversity estimated by Nonpareil 3 compared to greywater samples (Figure 9A). Although soil samples resulted in higher sequence numbers, metagenomic coverage was lower than greywater samples. In addition, much smaller proportions of reads were mapped to ARGs in the CARD database based on RGI output (Figure 9B), contributing to the lower ARG relative abundances (RPKM) in soil samples. From metagenomic-based analyses, we did not observe the propragation of ARGs from treated greywater to the soil irrigated by it in the same household (See Table S4 Supplementary Materials). However, qPCR detected the same ARGs in treated greywater and soil samples. It is likely that due to a lack of sequencing effort covering the more diverse soil microbial communities, we were not able to capture whole ARG profiles with the soil metagenomes.

The relative abundance of ARG in soil samples is very low after filtering out low-quality mapping results. Considering the high variation and complexity of soil microbial communities [82,83], more studies are needed to fill the knowledge gap regarding the influence of treated greywater irrigation on ARG prevalence in soils and soil microbial communities [68,84]. Soil texture and properties such as clay and soil organic matter contents are more important drivers than irrigation water (potable water or treated wastewater) in shaping the diversity and composition of soil microbial communities.

## 3. Materials and Methods

All the RVFCW systems are located in the village Midreshet Ben Gurion, southern region of Israel (coordinates: 30°51′8″ N 34°47′0″ E) and have been operating for over ten years. The households received freshwater from the same source and have a few different setup parameters or conditions, as listed in Table 3. Raw and treated greywater samples were collected periodically in 2021–2022 during the months of June (summer) and December (winter). Raw greywater samples were collected from the sewage basin, and treated greywater samples were collected from the lower container. Overall, 14 samples of 1L raw greywater samples and 15 samples of 5 L treated greywater samples were collected. In winter 2022, samples of the filter bed were collected from the wetland bed in the upper container at 10 cm depth in small sterile bottles.

Soil samples irrigated with potable water or treated greywater were collected during winter 2021 before the rainy season. The samples were collected randomly next to the irrigation drippers at 0–5 cm depth.

The samples were collected and transported to the laboratory within 4 h. The greywater samples were stored at 2 °C and filtered within 24 h for DNA extraction. Physicochemical parameters of the samples were assessed, also within 24 h. Electrical conductivity and pH were measured using a CyberScan510 pH meter (Eutech Instruments, Thermo, Waltham, MA, USA). Total organic carbon (TOC) and total nitrogen (TN) were quantified using a Multi N/C^®^ 2100S analyzer (Analytik Jena AG, Jena, Germany). Total suspended solids (TSS) and five-day biological oxygen demand (BOD) were determined according to standard analytical methods for the examination of water and greywater by following the standard procedure [85].

### 3.1. DNA Extraction and qPCR

The collected raw and treated greywater samples were filtered with a glass fiber filter (GF type A/E 46 mm PALL) and 0.2 µm filter (PALL). Filterbed media was vortexed and centrifuged with a sterile phosphate-buffered saline solution (Sigma (St. Louis, MO, USA), P4417-100TAB). Total genomic DNA was extracted from the filters using the DNeasy PowerWater Kit (Qiagen (Hilden, Germany), product code 14900-100-NF). DNA concentrations were determined using the NanoDrop 1000 instrument (NanoDrop Technologies, Wilmington, DE, USA) and analyzed by PCR and quantitative PCR (qPCR) assays to quantify the abundance of bacterial and antibiotic resistance genes. For all samples, PCR and qPCR were performed with 20 µL reaction volumes composed of 10 µL Ready-Mix for PCR (cat. 9597 58026540Biolab, Jerusalem, Israel) and qPCRBIO SyGreen blue mix lo-rox (PCR biosystems, London, UK), 1.6 µL of the respective primers (forward and reverse), 7.4 µL of nuclease-free water, and 1 µL of template DNA. The PCR and qPCR cycling conditions for all reactions were as follows: 30 cycles composed of 5 min denaturation at 95 °C; 1 min annealing at 60 °C; and 30 s polymerization at 72 °C. The amplicons were analyzed by gel electrophoresis (in 2% agarose in TAE buffer), stained with ethidium bromide, and visualized under UV light. Based on the results of the PCR analysis, the genes *blaTEM*, *sul1*, *intI1*, *tetG*, *tetW*, *FabVas*, and *FabVas2* were selected for the qPCR analysis. It was performed in triplicate with a calibration curve and no template control (NTC). The calibration curves were built using an eight-fold dilution series of synthetic plasmid pNORM containing synthetic inserts of genes targeting different classes of antimicrobials [86]. The complete gene copies of target genes were calculated based on known copies of standard reference plasmid. Relative gene abundance was calculated by normalizing the absolute number of ARG copies to 16S rRNA gene copies.

### 3.2. Detection of Micropollutants by LCMS

Collected soil samples (about 10 g) were mixed individually with 10 mL of acetonitrile and were extracted using the QuEChERS method [87]. The content of an ECQUEU750CT-MP Mylar pouch (United Chemical Technologies, Levittown, PA, USA) was added to each sample, shaken immediately for more than 2 min, and centrifuged for 5 min at ≥3000 rcf. The supernatant was filtered directly into a sample vial through a 0.2 μm PVDF syringe filter. Ten microliters of each sample were injected into the Liquid Chromatography Mass-Spectrometry (LCMS) instrument [88], consisting of a 1260 Infinity II pump (model G7111B) coupled to a triple quadrupole (model G6465B) mass spectrometer with an Electrospray Ionization source (Agilent Technologies Inc., Santa Clara, CA, USA). The column was Kinetex XB-C18, 3.0 mm × 100 mm, 2.6 μm (Phenomenex, Torrance, CA, USA). The flow rate was 0.2 mL/min, and the gradient was as follows: 1 min at 50% MeOH; 5 min increase to 95% MeOH; 3 min at 95% MeOH; 1 min decrease to 50% MeOH; 1 min 50% MeOH.

### 3.3. SmartChip™ Analysis

Samples for Resistomap were collected during November 2022 using the same sampling method as described above. ARGs, mobile gene elements (MGEs), and 16S rRNA genes in each sample, originating from raw greywater, treated greywater, and filter bed, were analyzed using customized primer sets in a high throughput method, SmartChip™ qPCR system (Resistomap Oy, Helsinki, Finland). The qPCR reactions were performed using 36 primer sets (Table S1, Supplementary Materials), selected based on frequent ARGs and MGEs previously detected globally [89,90]. Several primer sets were designed to target sequence diversity within the gene target to assess the environmental resistome more precisely; therefore, each primer set was analyzed independently [91,92,93].

### 3.4. Metagenomics

DNA extracts were sent to the NUSeq Core Facility at Northwestern University for shotgun metagenomic DNA sequencing. Illumina DNA Prep Kit was used for sequencing library construction, and an Illumina HiSeq 4000 platform was used for paired-end 150-bp sequencing. Decontamination of sequences was performed with KneadData v0.7.4 ([94]) using the default settings to eliminate human genome and sequences from two kit controls (namely, Qiagen glass filter and PALL and Lucigen glass filter and PALL) samples. Sequence quality was checked before and after decontamination using MultiQC v1.10.1 [95]. Decontaminated and paired reads from Kneaddata were used to estimate metagenomic coverage and sequence diversity by Nonpareil 3 (v3.304) [96]. Metagenomics, short read-based ARG identification was performed using Resistance Gene Identifier (RGI version 5.2.1; https://github.com/arpcard/rgi, accessed on 4 January 2022) bwt mode from the Comprehensive Antibiotic Resistance Database (CARD) using database version 3.1.2 [97]. The ARG-mapped reads from RGI were filtered so that only MAPQ ≥ 50 and coverage ≥ 90% of the reference length or read length (150 bp) were attained. The retained mapped read counts were then converted to the relative abundance of each ARG allele in the unit of reads per kilobase per million mapped reads (RPKM), which allows for comparison between samples.

### 3.5. Statistical Analysis

Significance (*p* < 0.05) was assessed using the *t*-test in R software. To visualize data, we used the ggplot2 package in R software. All statistical analyses were performed in R software v3.6.2 unless otherwise stated.

## 4. Conclusions

The current study investigates for the first time the potential development of ARGs within RVFCWs and the potential dispersion of ARGs in the gardens irrigated by the effluent of RVFCWs. We analyzed greywater and soil samples for antimicrobial chemicals, microbial community profiles, and antibiotic resistance gene profiles. Antibiotic resistance genes are ubiquitous, present albeit at low levels in human-associated environments and even drinking water. It is, therefore, unreasonable to expect a complete absence in either treated greywater or irrigated soil [98,99]. However, based on the abundance, diversity, and presence of specific genes of concern, we can weigh the risks of greywater use for irrigation and the benefits of specific technologies.

(1) RVFCWs performance in relation to ARGs is highly variable: qPCR analysis presented that the total bacterial abundance was reduced in most households after treatment. In the winter season, most of the ARGs were significantly decreased after treatment, specifically for genes *tetG*, *sul1,* and *blaTEM*. Resistomap analysis analyzed 34 genes simultaneously; filter bed samples had the most microbial load compared to the raw and treated greywater samples. Genes conferring resistance to aminoglycoside, beta-lactam, vancomycin, and quinolone were increased in treated greywater;
(2) Overall risks and impact on soil are low: The type of irrigation water (potable or treated greywater) had no specific influence on the soil’s total bacterial abundance (16S rRNA gene). No overlapping ARGs were found when tracing from treated greywater to soil irrigated with treated greywater;
(3) More characterization of these systems will better reveal how they work, enabling more robust design and ensuring risks stay low: Future research should assess factors that modify the effect of wastewater irrigation on ARGs in soil. High throughput qPCR and metagenomics should be used to comprehensively evaluate ARGs in different soil types. Moreover, shotgun sequencing of the filter bed could give an insight into changes in the catabolic pathways and enzymes with the potential for biodegradation of micropollutants.

## Figures and Tables

**Table 1:** The average level of raw greywater parameters of all households together. The values of raw greywater are measured during 2021 and for treated greywater during 2015–2021.

Parameter	Raw Greywater	Treated Greywater
pH	6.95–9.63	6.1–10.16
EC [µs/cm]	366–2150	321–995
Turbidity [NTU]	15.07–271.7	0–68
BOD [mg/L]	10.9–39.9	0.45–21.9
TSS [g/L]	12.43–26.92	0.001–47
TOC [mg/L]	5.47–43.78	1.68–39.1
TN [mg/L]	1.72–25.13	0.4–38.3

**Table 2:** The maximum absolute abundance values of each gene that were detected in raw, treated, and filter beds.

Gene	Abundance	Sample Type	Gene	Abundance	Sample Type
*intI1_1*	8.73 × 10^6^	Filterbed	*aac(6′)-II*	1.27 × 10^4^	Filterbed
*vanA*	2.24 × 10^6^	Filterbed	*aph6*	2.22 × 10^4^	Raw
*vanXD*	2.43 × 10^3^	Treated	*blaCTX-M*	5.96 × 10^5^	Filterbed
*ermD*	2.03 × 10^3^	Filterbed	*blaCMY2*	3.05 × 10^5^	Filterbed
*mcr1_1*	1.92 × 10^6^	Filterbed	*blaOXA51*	1.32 × 10^4^	Filterbed
*qacE*	1.33 × 10^6^	Raw	*adeA*	4.14 × 10^5^	Raw
*fabK*	1.12 × 10^4^	Filterbed	*pcoA*	5.27 × 10^4^	Filterbed
*ISPps*	2.47 × 10^6^	Filterbed	*qnrB*	7.45 × 10^4^	Filterbed
*tnpA_1*	6.00 × 10^5^	Filterbed	*qnrD*	1.12 × 10^4^	Filterbed
*Tn3*	1.64 × 10^4^	Raw	*cmlA_2*	6.09 × 10^4^	Filterbed
*sul2_1*	3.50 × 10^6^	Treated	*catB3*	4.94 × 10^4^	Treated
*sul1_2*	9.19 × 10^5^	Treated	*ermF*	9.56 × 10^4^	Filterbed
*aph3-ib*	1.39 × 10^6^	Filterbed	*erm34*	2.06 × 10^4^	Filterbed
*aadA_1*	7.48 × 10^5^	Raw	*ermX_1*	4.90 × 10^3^	Filterbed
*vgaA_1*	5.94 × 10^3^	Filterbed	*dfrA1*	9.52 × 10^4^	Raw
*tetA_1*	4.09 × 10^3^	Filterbed	*tetW*	1.51 × 10^4^	Treated
*tetC_1*	1.16 × 10^3^	Filterbed			

**Table 3:** Different setup parameters and conditions of the five households using the RVFCW system.

	Filter Bedding	Vegetation	Special Remarks
House 1	Small tuff stone	*Iris pseudacorus*	
House 2	Big tuff stone	*Nastrurtium officinale* and *Equisetum hyemale*	
House 3	Mainly big pebbles with some small tuff stones	No vegetation	The upper container is completely covered with a lid
House 4	Small tuff stone	*Iris pseudacorus*	The system also receives water from the house swimming pool
House 5	Plastic beads and some small tuff stones	*Iris pseudacorus*	
House 6	Gravel stones	*Solanum lycopersicum*	
House 7	Small tuff stone	*Canna indica*	

## Data Availability

Raw PCR data will be available from the main author upon request.

## Supplementary Materials

The following supporting information can be downloaded at https://www.mdpi.com/article/10.3390/antibiotics13040315/s1, Figure S1: Recirculating vertical-flow constructed wetland. Figure S2: Relative abundance of antimicrobial resistance genes in greywater samples collected from different families. Data are shown on a log scale as relative gene copies normalized to the copies of 16S rRNA genes from each sample. Figure S3: The relative abundance of ARGs for each sample, clustered according to each household. Figure S4: Heatmap showing the composition of gene abundances relative to the 16S rRNA gene. The color bar on the right means relative abundance from low (blue) to high (red) levels. Table S1: Details of selected primers used for SmartChip™ analysis. Table S2: Average level of treated greywater parameters during 2015–2021, shown for each household separately. Table S3: Correlation between ARGs and emerging micropollutants as detected in soil samples. Numbers in bold indicate a relationship. Table S4: Short read-based antimicrobial resistance gene identification.
