# Supplementary material for: Heterogeneous Antibiotic Resistance Gene Removal Impedes Evaluation of Constructed Wetlands for Effective Greywater Treatment
Source: Antibiotics (Basel). 2024 Mar 29;13(4):315. doi: 10.3390/antibiotics13040315 (PMC11047525; doi:10.3390/antibiotics13040315)
Supplement: Supplementary file 1 [file antibiotics-13-00315-s001.zip › antibiotics-2924016-supplementary.pdf]

antibiotics

Supplementary Materials

RVFCW system characteristics

The RVFCW is a decentralized system consisting of two 500 L capacity stacked containers. The upper container holds a constructed wetland bed. Raw GW is introduced directly into the plant root zone, from where it trickles through the passively aerated wetland bed. Further aeration takes place as the water drops into the lower container reservoir. From the reservoir, the water is recirculated back to the wetland bed for 6–8 hours, after which it is pumped out for landscape irrigation, either with or without a disinfection stage.

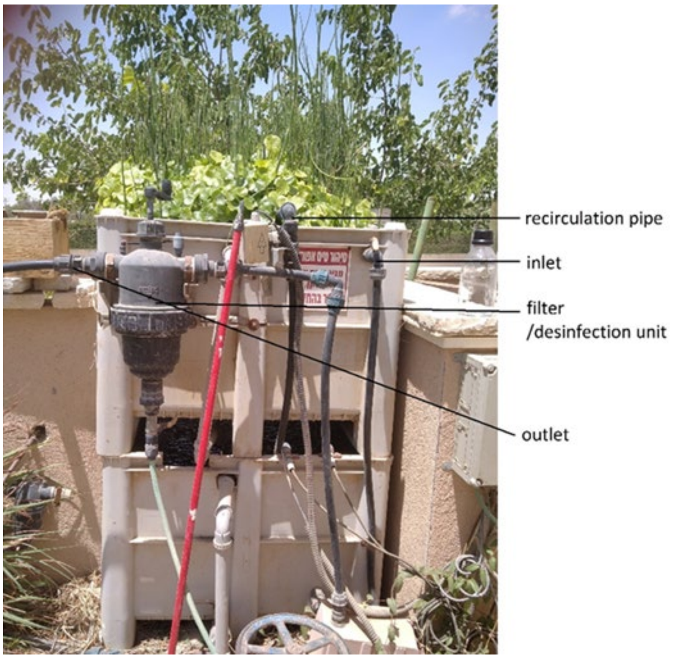

Figure S1. Recirculating vertical-flow-constructed wetland.

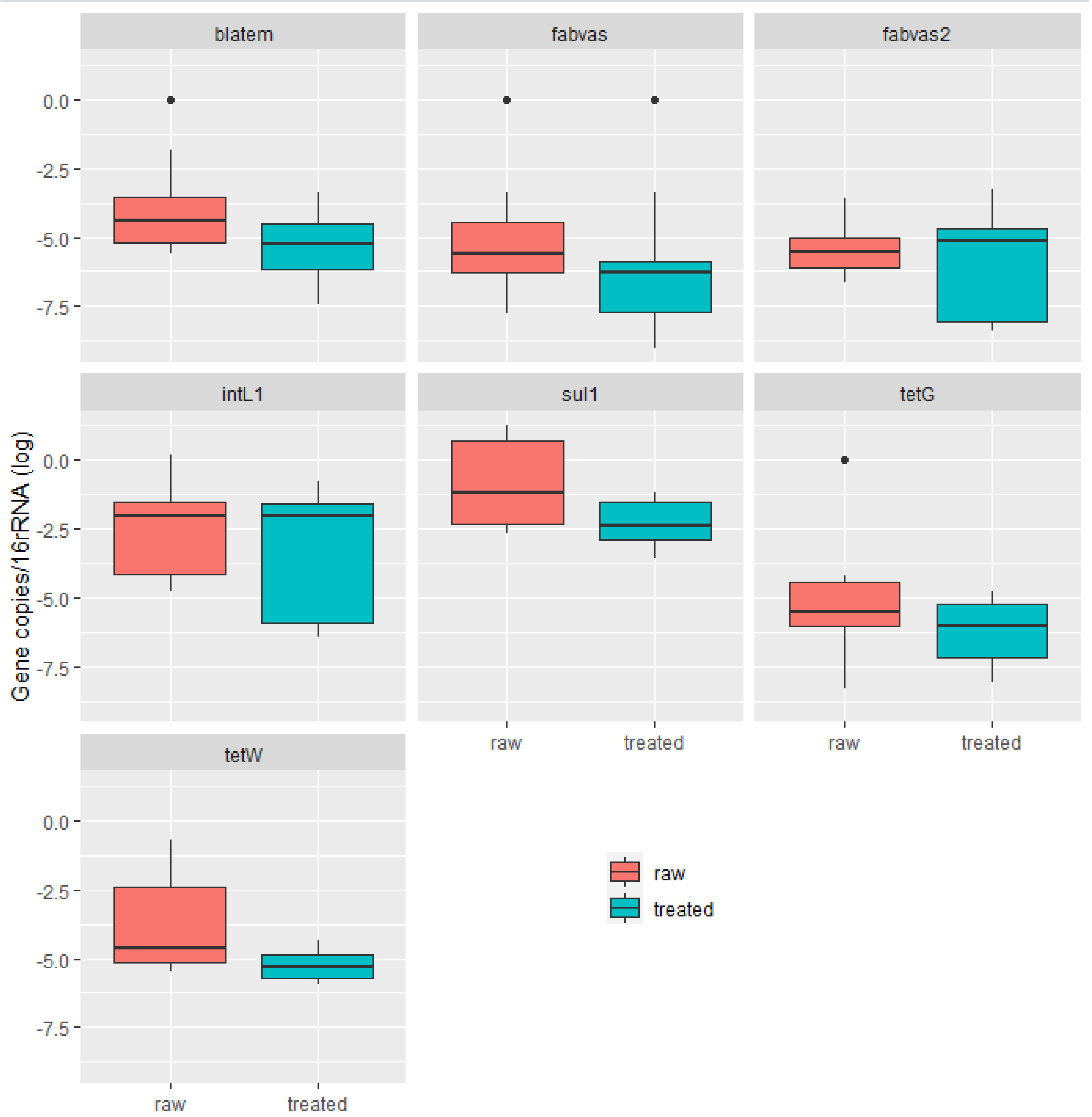

Figure S2. Relative abundance of antimicrobial resistance genes in greywater samples collected from different families. Data are shown on a log scale as relative gene copies normalized to the copies of 16S rRNA genes from each sample.

Table S1. Details of selected primers used for SmartChip™ analysis.

Assay	Gene	Target Antibiotics (Major)	Forward Primer	Reverse Primer
AY1	16S rRNA	16S rRNA	GGGTTGCGCTCGTTGC	ATGGYTGTCGTCAGCTCGTG
AY6	aac(6')-II	Aminoglycoside	CGACCCGACTCCGAACAA	GCACGAATCCTGCCTTCTCA
AY10	aadA_1	Aminoglycoside	GTTGTGCACGACGACATCATT	GGCTCGAAGATACCTGCAAGAA
AY419	aph3-ib	Aminoglycoside	AACAGGTTTGGGAGGCGATG	CGCAACAAGCCTCTCCTGAA
AY326	aph6	Aminoglycoside	CCCATCCCATGTGTAAGGAAA	GCCACCGCTTCTGCTGTAC
AY116	blaCMY2	Beta Lactam	GCGAGCAGCCTGAAGCA	CGGATGGGCTTGTCCTCTT
AY432	blaCTX-M	Beta Lactam	CGTACCGAGCCGACGTTAA	CAACCCAGGAAGCAGGCA
AY435	blaOXA51	Beta Lactam	CGACCGAGTATGTACCTGCTTC	TCAAGTCCAATACGACGAGCTA
AY293	intI1_1	Integrons	CGAACGAGTGGCGGAGGGTG	TACCCGAGAGCTTGGCACCCA
AY202	adeA	MDR	CAGTTCGAGCGCCTATTTCTG	CGCCCTGACCGACCAAT
AY206	cmr	MDR	CGGCATCGTCAGTGGAATT	CGGTTCCGAAAAAGATGGAA
AY495	pcoA	MDR	TGGCGTATGGAGTTTCAATGC	GAATAATGCCGTGCCAGTGAA
AY309	ISPps	MGE	CACACTGCAAAAACGCATCCT	TGTCTTTGGCGTCACAGTTCTC
AY523	Tn3	MGE	GCTGAGGTGTTCAGCTACATC C	GCTGAGGTAGTCACAGGCATTC
AY299	tnpA_1	MGE	GCCGCACTGTCGATTTTTATC	GCGGGATCTGCCACTTCTT
AY530	erm34	MLSB	AAAGCGGTTTACAAGCGTTTC G	GGGTGCTCTAGGGTTGTTTAGTG
AY534	ermD	MLSB	TTTCCGGACAGCATTTGATGC	TCCACTGCCAATACCTTACCG
AY535	ermF	MLSB	TCTGATGCCCGAAATGTTCAA G	TGAAGGACAATTGAACCTCCCA
AY68	ermX_1	MLSB	GCTCAGTGGTCCCCATGGT	ATCCCCCCGTCAACGTTT
AY71	vgaA_1	MLSB	CGAGTATTGTGGAAAGCAGCT AGTT	CCCGTACCGTTAGAGCCGATA
AY472	fabK	Other	CAGGAGCAGGAAATCCAAGC	CCAGCTTCCATTCCTTCTGC
AY193	mcr1_1	Other	TCTTGTGGCGAGTGTTGCCGT	CCAATGATACGCATGATAAACG CTG
AY218	qacEΔ1_1	Other	TCGCAACATCCGCATTAAAA	ATGGATTTCAGAACCAGAGAAA GAAA
AY29	catB3	Phenicol	GCACTCGATGCCTTCCAAAA	AGAGCCGATCCAAACGTCAT
AY35	cmlA_2	Phenicol	TAGGAAGCATCGGAACGTTGA T	CAGACCGAGCACGACTGTTG
AY96	qnrB	Quinolone	GCGACGTTCAGTGGTTCAGA	GCTGCTCGCCAGTCGAA
AY459	qnrD	Quinolone	CGCTGGAATGGCACTGTGA	GCTCTCCATCCAACTTCACTCC
AY245	sul1_2	Sulfonamide	GCCGATGAGATCAGACGTATT G	CGCATAGCGCTGGGTTTC
AY243	sul2_1	Sulfonamide	TCCGGTGGAGGCCGGTATCTG G	CGGGAATGCCATCTGCCTTGAG
AY248	tetA_1	Tetracycline	GCTGTTTGTTCTGCCGGAAA	GGTTAAGTTCCTTGAACGCAAAC T
AY256	tetC_1	Tetracycline	CATATCGCAATACATGCGAAA AA	AAAGCCGCGGTAAATAGCAA

AY263	tetW	Tetracycline	ATGAACATTCCCACCGTTATCTTT	ATATCGGCGGAGAGCTTATCC
AY579	dfrA1	Trimethoprim	CGGATCATGTCATTGTTTCAGG	ATGTTAGAGGCGAAGTCTTGG
AY595	vanA	Vancomycin	GGGCTGTGAGGTCGGTTG	TTCAGTACAATGCGGCCGTTA
AY170	vanXB	Vancomycin	AGGCACAAAATCGAAGATGCTT	GGGTATGGCTCATCAATCAACTT
AY169	vanXD	Vancomycin	TAAACCGTGTTATGGGAACGAA	GCGATAGCCGTCCCATAAGA

Physicochemical parameters of greywater

This study analyzed physicochemical parameters for all raw and treated samples from different households to align with the microbial community. Values of raw GW are shown in Table 4, and values of the treated GW are displayed in Table 5. For raw GW, the homes were sampled twice in successive months; therefore, the values are shown for all five households together. The mean pH value is 7.64 for EC 856 μS/cm. The mean value of TSS is reported at 18.7 mg/L. The average BOD value in the samples is 28.4 mg/L, while the observed TOC value is 18 mg/L. The mean value of TN is detected at 9.8 mg/L. For treated GW, mean pH values ranged from 7.25 to 8.08, and values of EC obtained for the samples ranged from 459.9 to 659.7 μS/cm. Measured values of TSS varied considerably among the samples ranging from 1.1 to 19.1 mg/L. The average BOD values in the samples ranged from 2.9 to 10 mg/L, while observed TOC values ranged from 3.9 to 9.8 mg/L. The mean values of TN ranged from 2.6 to 14.9 mg/L. The values did not vary significantly among the different households and during the years, meaning the systems are stable and well-established.

Based on both tables, it can be concluded that the RVFCW system effectively removes chemical and biological contaminants. Overall, it reduces BOD and TSS by 80%, TOC by 60%, TN by 30%, and turbidity by 90%. These findings coincide with Gross et al. (2006), reconfirming that the RVFCW is a sustainable and promising treatment system for GW use

Table S2. Average level of treated greywater parameters during 2015–2021, shown for each household separately.

	**Household 1**		**Household 2**		**Household 3**		**Household 4**		**Household 5**	
parameter	range	mean ± st dev	range	mean ± st dev	range	mean ± st dev	range	mean ± st dev	range	mean ± st dev
pH	6.1–7.85	7.25 ± 0.52	6.63–9.92	7.67 ± 0.85	6.3–9.57	7.71 ± 0.96	6.65–8.12	7.47 ± 0.52	6.48–10.16	8.08 ± 1.05
EC [μs/cm]	346–995	578.25 ± 177.23	364–984	659.73 ± 191.67	329–993	638.3 ± 214.37	335–623	486.88 ± 104.27	321–671	459.91 ± 114.63
Turbidity [NTU]	0–66	19.14 ± 20.02	0–68	14.43 ± 20.41	0–43.2	8.21 ± 14.47	0–6.05	1.11 ± 2.1	0–13.52	2.42 ± 4.29
BOD [mg/L]	1.35–14.4	6.54 ± 3.56	0.45–9.99	4.51 ± 3.18	0.8–47	10 ± 14.7	0.72–6.6	2.95 ± 2.15	1.2–21.9	4.54 ± 6.05
TSS [g/L]	0.01–47	9.93 ± 15.81	0.001–47	5.83 ± 14.37	0.001–7	1.0 ± 2.16	0.001–2	0.25 ± 0.71	0.001–4	0.8 ± 1.3
TOC [mg/l]	4.56–17.8	9.61 ± 4.41	1.68–39.1	9.82 ± 11.91	2.74–21.74	7.61 ± 5.91	2.67–7.37	1.48 ± 3.95	2.9–9.68	5.26 ± 1.93
TN [mg/l]	1.32–12.85	6.92 ± 4.11	1.66–38.3	14.9 ± 10.11	0.62–9.72	3.7 ± 2.82	0.4–18.22	5.71 ± 5.91	0.42–6.63	2.57 ± 2.07

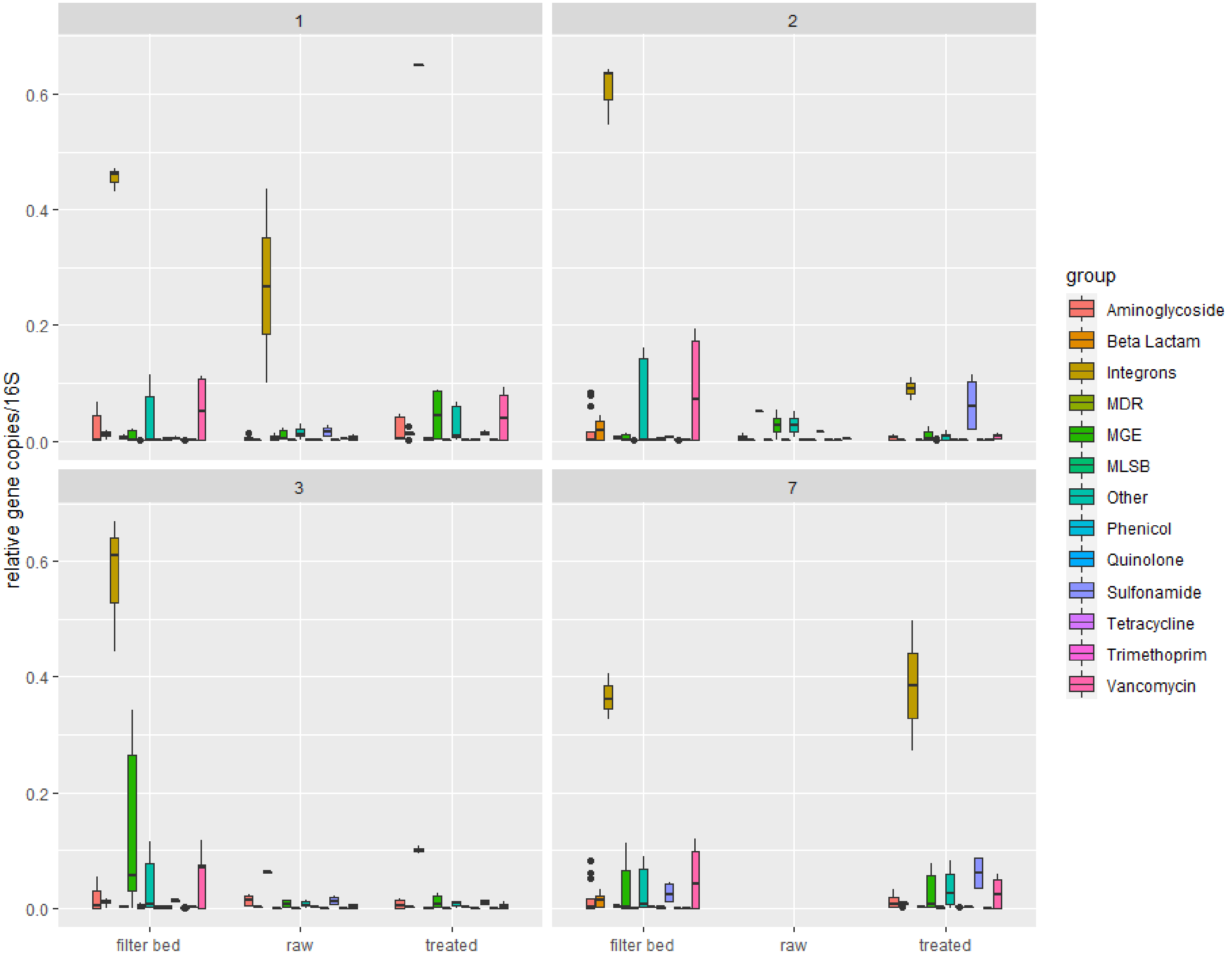

Figure S3. The relative abundance of ARGs for each sample, clustered according to each household.

Table S3: Correlation between ARGs and emerging micropollutants as detected in soil samples. Numbers in bold indicate a relationship.

	fabvas2	*tetW*	*tetG*	*blatem*
Ethylparaben	0.35	0.10	-0.47	-0.26
Methylparaben	**0.55**	0.33	**-0.69**	-0.25
Octocrylene	-0.22	-0.26	0.46	-0.21
Propylparaben	0.35	0.15	**-0.51**	-0.21
Sulfamethoxazole	**0.59**	0.37	-0.53	-0.19
Tonalid	-0.17	-0.16	-0.35	-0.49
Triclosan	0.35	0.15	-0.51	-0.21

The results showed that of the observed seven micropollutants in the soil, only three had a correlation with the detected genes, of which none had a strong relation. The gene *fabvas2* has a positive correlation with methylparaben and sulfamethoxazole, and the gene *tetG* has a negative correlation with methylparaben and propylparaben.

Table S4. Short read-based antimicrobial resistance gene identification.

**House 1**	**House 1**	**House 2**	**House 2**	**House 3**	**House 3**
**treated greywater-irrigated soil**	**greywater-irrigated soil**	**treated greywater-irrigated soil**	**greywater-irrigated soil**	**treated greywater-irrigated soil**	**greywater-irrigated soil**
Acinetobacter baumannii AbaQ		AER-1		ACI-1	
OpmH		CAM-1		CRP	
Txr		LRA-19		dfrA22	
adeF	adeG	*Pseudomonas aeruginosa* soxR		mel	
sul2		adeF		ramA	
tet(49)		adeJ		sul4	
vanHF	vanXI	adeK		tet(A)	tet(56)
		mphC			ErmY
		tet(A)			vanXF
			vanF		

Overlapping ARGs across soil samples are rare; this trend is similar to the greywater samples. Tracing from treated greywater to greywater irrigated soil in the same household, no overlapping ARGs were found.

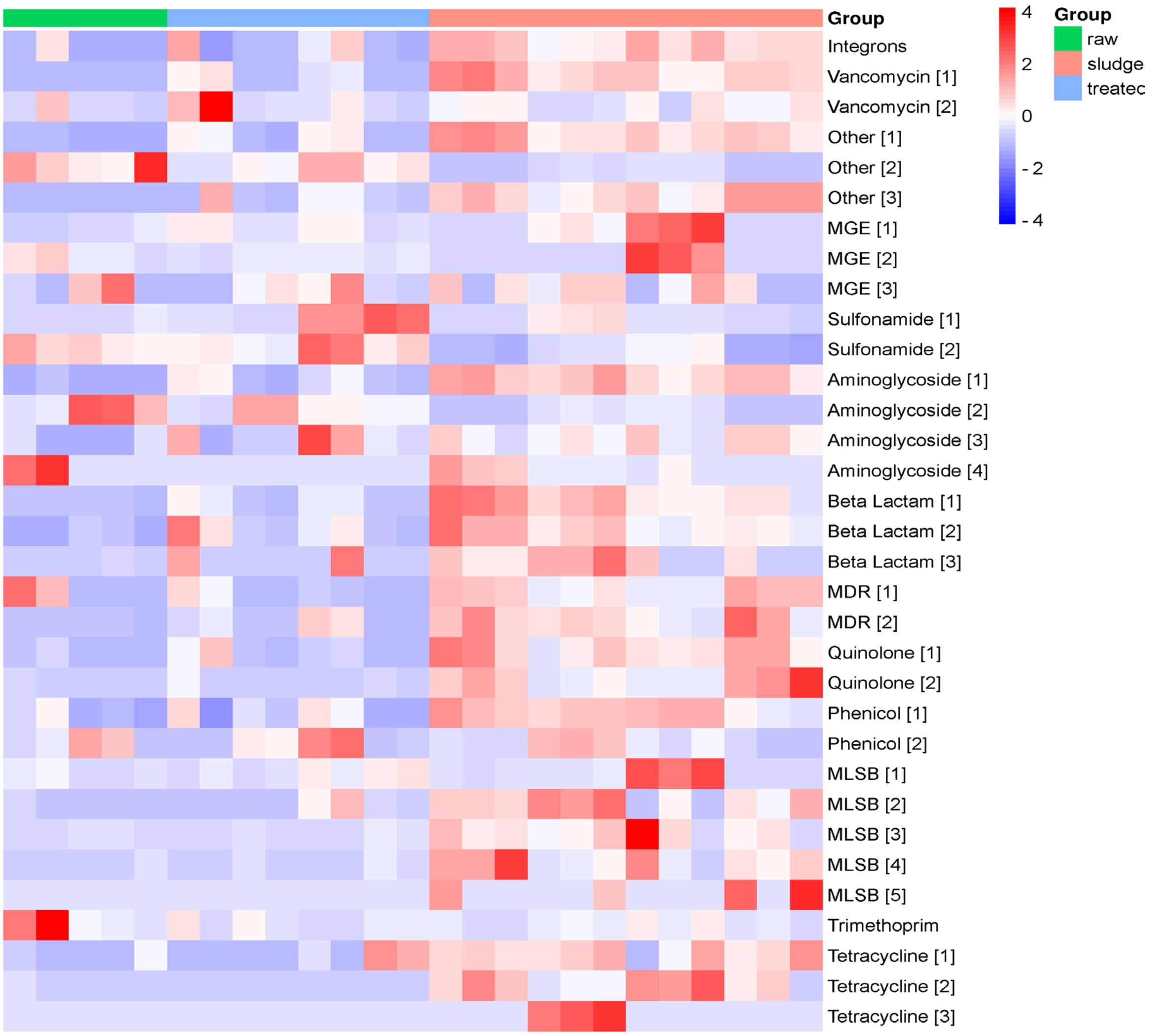

Figure S4. Heatmap showing the composition of gene abundances relative to the 16S rRNA gene. The color bar on the right means relative abundance from low (blue) to high (red) levels.
